# Cannabis Butane Hash Oil Dabbing Induced Lung Injury Mimicking Atypical Pneumonia

**DOI:** 10.7759/cureus.7033

**Published:** 2020-02-18

**Authors:** Danica Stephens, Jinal K Patel, Debra Angelo, Johnathan Frunzi

**Affiliations:** 1 Internal Medicine, Medical Center of Trinity, Odessa, USA; 2 Internal Medicine, Medical Center of Trinity, Trinity, USA

**Keywords:** dabbing and lung injury

## Abstract

“Dabbing” is the inhalation of concentrated marijuana, usually in butane solvent. This case report illustrates a previously healthy 25-year-old caucasian male with a 10-year history of cannabis butane hash oil (BHO) use. The patient presented with dyspnea and cough. The evaluation included a chest x-ray, basic laboratory investigations, computerized tomography angiogram of the chest and echocardiogram. Patient was diagnosed with acute lung injury mimicking atypical pneumonia. He was treated with steroids and had clinically improved and advised to stop dabbing. Further studies are needed to elucidate the full spectrum of the adverse effects of dabbing.

## Introduction

“Dabbing” is a form of marijuana use that enables inhalation of high concentrations of tetrahydrocannabinol (THC) by heating butane hash oil (BHO) on a titanium nail mounted on a water pipe [1]. The nail is heated with a gas torch (which does not allow adequate temperature control) until glowing red, then cooled for a few seconds. A small amount of BHO is then placed on the hot surface, where it quickly vaporizes. The resulting condensate is then inhaled deeply in a single puff and held in the lungs for several seconds [1-3]. Here, we present a case report of an adult with chronic inhalation of vaporized BHO-induced lung injury mimicking atypical pneumonia. 

## Case presentation

A 25-year-old caucasian male, with no past medical history presented to the emergency department with shortness of breath and cough that began three weeks prior to presentation. The patient stated his initial symptoms were congestion and dry hacking cough associated with yellow-colored sputum. The symptoms had progressively worsened, and he had only minimal improvement with over-the-counter medication. He denied fever, chest pain, and hazardous material exposure; however, he stated that he has been “dabbing” on a daily basis for ten years.

**Figure 1:**
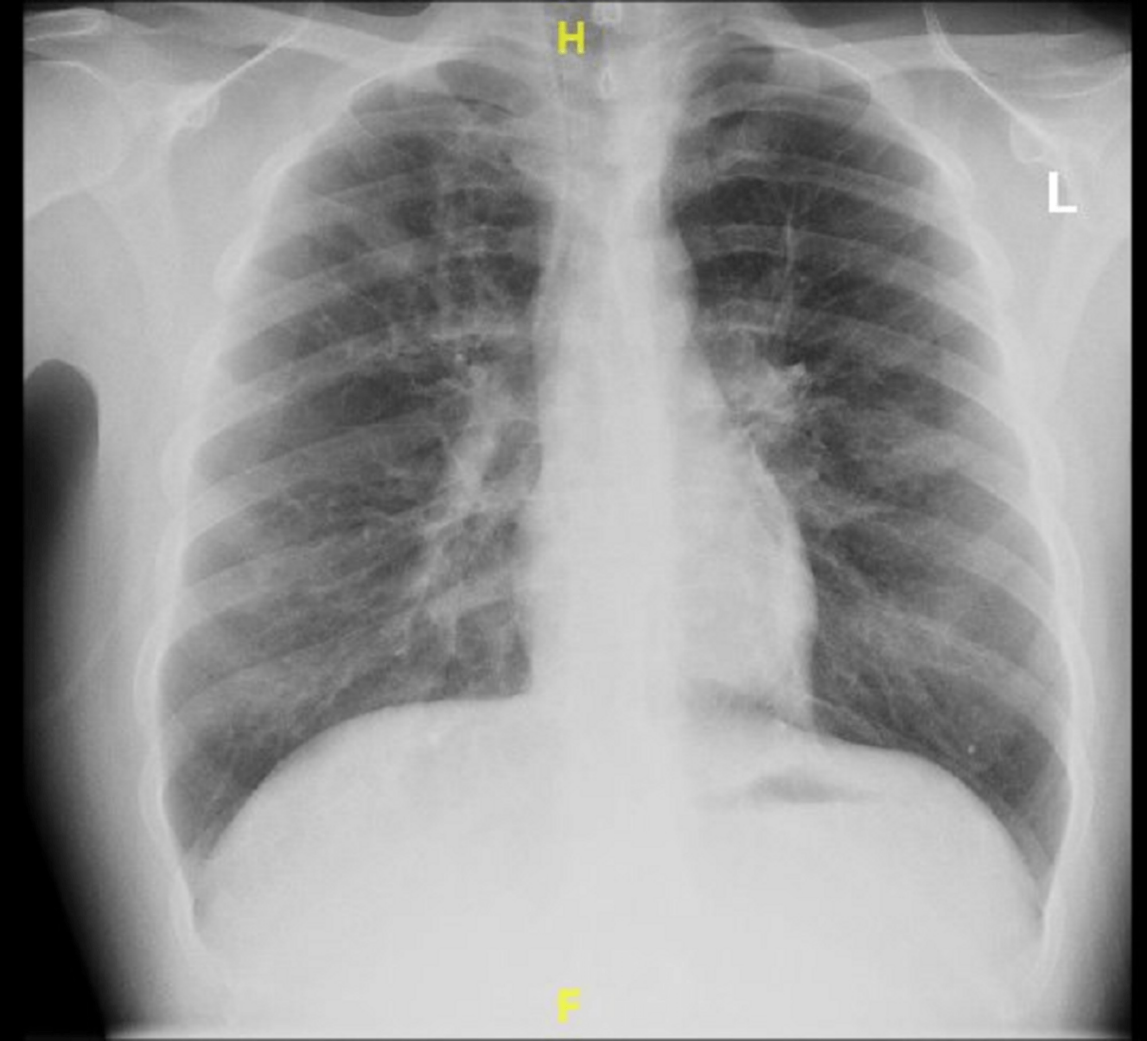
Posterioanterior Chest X-ray No acute findings

**Figure 2:**
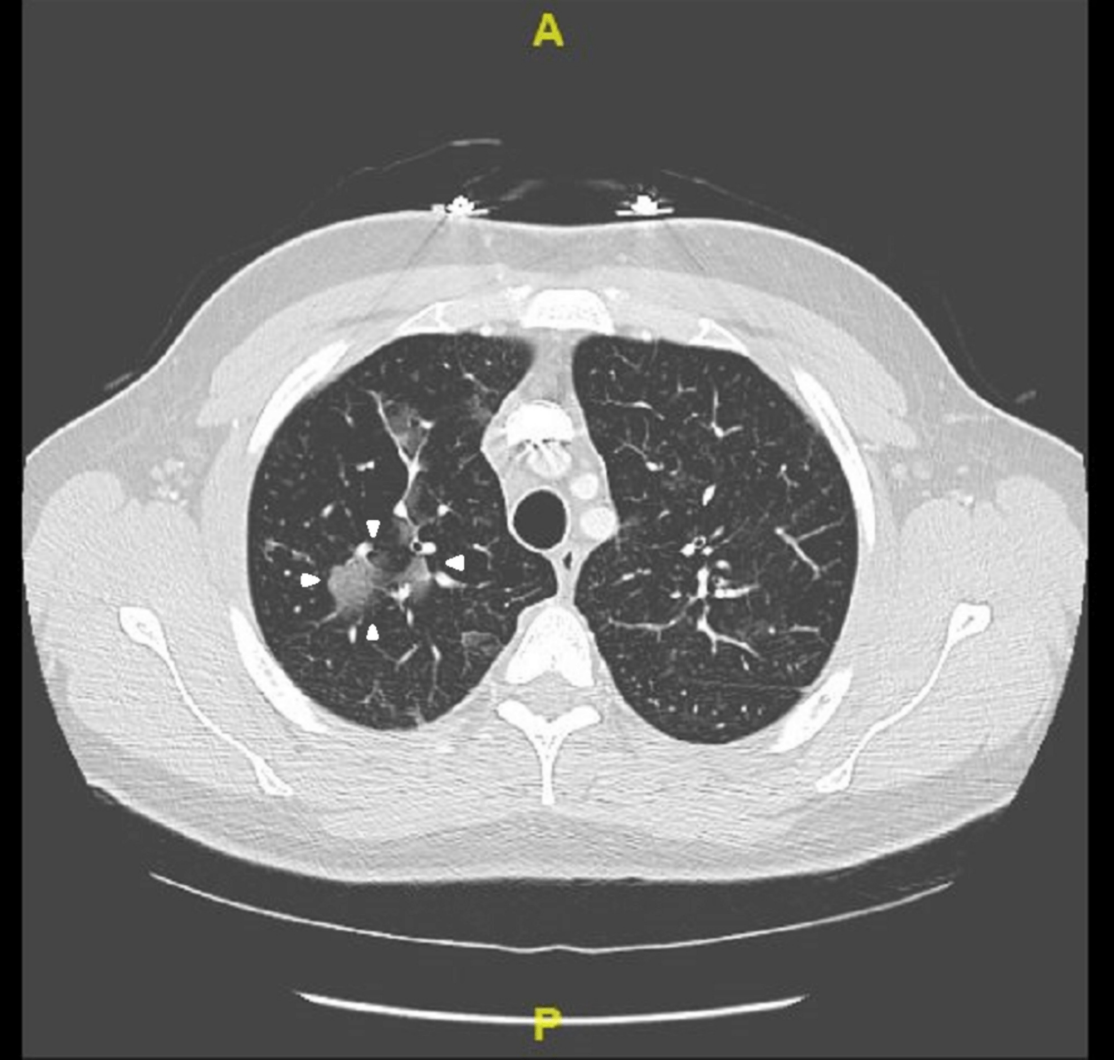
CT Angiogram of Chest with contrast

**Figure 3:**
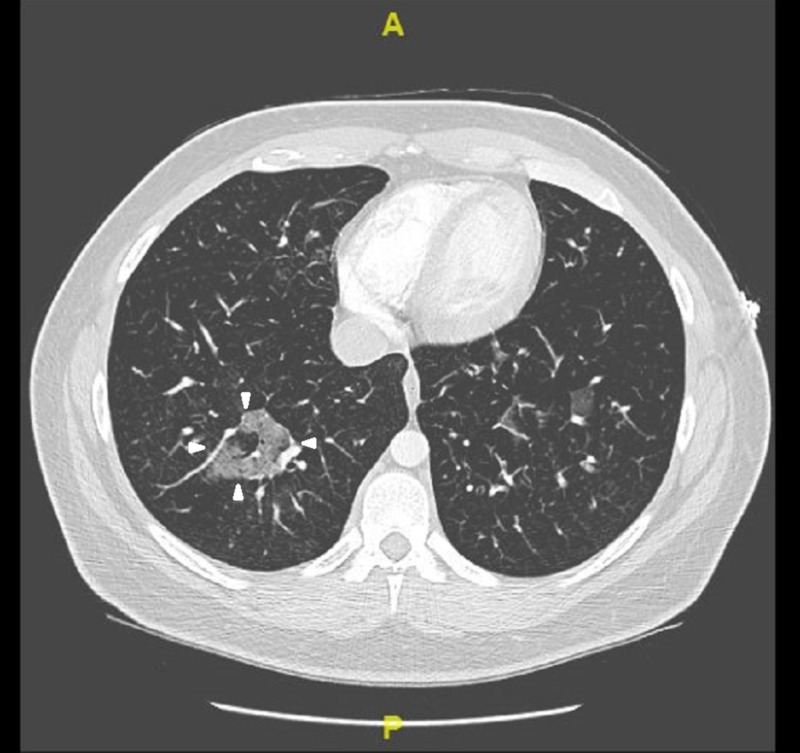
CT Angiogram of Chest with contrast

**Figure 4:**
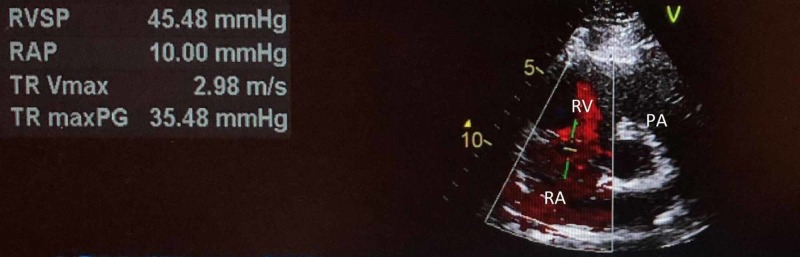
Echocardiogram Right Atrium (RA), Right Ventricle (RV), Pulmonary Artery (PA), Right Ventricle Systolic Pressure (RVSP)

On presentation, the patient was afebrile (98.1F), tachycardic at heart rate of 121 beats per minute and saturating at 88% on room air. Initial laboratory results revealed mild leukocytosis with white blood cell count of 11.32 K/mcL with isolated eosinophilia of 3.03 K/mcL. Respiratory exam revealed diffuse wheezing, crackles, and rhonchi bilaterally. On hospital day 2, the patient developed audible squeaking of the lungs associated with sputum change from initial presentation to white pearl-like with dark specks. Work-up included chest x-ray which did not show any acute changes (Figure 1). Computerized tomography (CT) angiogram of the chest showed multiple small-to-moderate polygonal foci of ground-glass infiltrates seen in all lobes, but greatest in the right upper and lower lobes (Figures 2, 3). Echocardiogram revealed moderate systolic pressure increase in pulmonary artery, with an estimated peak pressure of at least 45 mmHg (Figure 4). The patient was diagnosed with acute hypoxic respiratory failure secondary to chemical pneumonitis from BHO inhalation. He was treated with breathing treatments, oxygen with FiO2 of 36% via nasal cannula, empiric ceftriaxone and azithromycin, and 125 mg of intravenous (IV) prednisone. The IV prednisone was converted to oral prednisone 30mg/day after the first dose. 

## Discussion

Dabbing is a relatively novel method of ingesting cannabis that involves the use of highly concentrated THC in the form of butane hash oil at a poorly regulated high temperature. Lack of temperature control likely results in temperatures that lead to degradation of BHO terpenes to methacrolein and benzene [3]., Methacrolein is similar to acrolein, a pulmonary irritant which causes pulmonary edema and acute lung injury in laboratory animals [1,3]. Benzene is a known carcinogen which can lead to leukemia and bleeding disorders [4]. 

On literature review we were able to find two case reports related to dabbing and acute lung injury [3, 5]. The first case report, by McMahon and colleagues, describes a 19-year-old male who developed severe acute pneumonitis post deep BHO inhalation [5]. CT of the chest revealed diffuse bilateral infiltrates. He was initially treated with empiric antibiotics and intubated for acute hypoxemic respiratory failure. Later, he was extubated and clinically improved with supplemental oxygen and steroids [5]. The second case report, by Anderson and Zechar, describes an 18-year-old female with a 3-year history of BHO use who was diagnosed with severe pneumonitis with acute hypoxic respiratory failure secondary to BHO inhalation. The CT angiogram of her chest showed bilateral patchy infiltrates. Similar to the the first case, she showed clinical improvement with steroids [3]. 

Our case involved a 25-year-old male with similar findings and BHO inhalation method as the cases described above. His chest X-ray was non-contributory (Figure 1). CT angiogram of the chest revealed ground glass opacities, which were not found in the cases described above (Figures 2-3). Our patient was treated for presumed atypical pneumonia with ceftriaxone and azithromycin, as he did not initially disclose his marijuana use. Similar to the aforementioned cases our patient clinically improved with steroids and breathing treatments. However, our patient did not require intubation since he did not have severe respiratory failure as the first case described above [2]. Although not reported in either of the cases, our patient’s echocardiogram revealed elevated pulmonary artery pressure likely due to his chronic history of dabbing (Figure 4) [3,5].

Acute psychosis has also been reported in patients who inhaled high potency cannabis [6]. Our patient did not show any signs of acute psychosis after a reported ten-year history of daily dabbing.

## Conclusions

Our case adds to the limited body of evidence regarding the use of cannabis concentrates. The lack of knowledge in the general population about lung injury risks associated with dabbing and its widespread use will likely lead to an increase of patients presenting to hospitals with similar conditions. We recommend that clinicians obtain route of administration when obtaining a history from a patient with marijuana use to broaden the differential diagnosis and decrease antibiotic resistance. 
